# Evaluation of Serological Tests for the Diagnosis of Syphilis

**DOI:** 10.7759/cureus.61007

**Published:** 2024-05-24

**Authors:** Clarissa J Lyngdoh, Mandira Ramudamu, Manika Agarwal, Shikha Verma, Abhijit Prasad

**Affiliations:** 1 Microbiology, North Eastern Indira Gandhi Regional Institute of Health and Medical Sciences (NEIGRIHMS), Shillong, IND; 2 Microbiology, Bhaarath Medical College and Hospital, Chennai, IND; 3 Obstetrics and Gynaecology, North Eastern Indira Gandhi Regional Institute of Health and Medical Sciences (NEIGRIHMS), Shillong, IND; 4 Dermatology, North Eastern Indira Gandhi Regional Institute of Health and Medical Sciences (NEIGRIHMS), Shillong, IND; 5 Microbiology, All India Institute of Medical Sciences, Raipur, Raipur, IND

**Keywords:** hemagglutination assay, rapid plasma reagin test, non-treponemal test, treponemal test, syphilis

## Abstract

Background

Syphilis remains a significant public health concern in India. Ensuring the accuracy of diagnostic tests is crucial for effectively managing this disease.

Objectives

This study aims to assess the detectability of syphilis using commercially available non-treponemal and treponemal tests due to observed discrepancies in test results, which can lead to confusion and anxiety among healthcare providers and patients.

Materials and methods

We analyzed 2312 serum samples using the rapid plasma reagin (RPR), *Treponema pallidum* hemagglutination assay (TPHA), enzyme-linked immunosorbent assay (ELISA), and modified TPHA rapid test, interpreting the results according to the manufacturers' instructions. We evaluated the diagnostic accuracy of all four tests. Concordance between the traditional and reverse algorithms was determined by calculating the percentage of agreement and the kappa (κ) coefficient.

Results

Of the 2312 samples tested, 34 (1.5%) were positive, and 2098 (90.7%) were negative across all four tests. Comparing the test results with clinical diagnosis, TPHA and TP-ELISA showed the highest sensitivity at 96.08%, while RPR demonstrated the highest specificity at 100%. The agreement between the traditional and reverse algorithms was moderate, with a 97.3% agreement and a κ value of 0.53.

Conclusion

Reliance on a single serological test for syphilis screening presents limitations. A combined approach using both RPR and TPHA tests can more accurately diagnose and confirm syphilis. This combination strategy is cost-effective and relatively simple to implement.

## Introduction

Syphilis, caused by *Treponema pallidum*, affects approximately 12 million people annually worldwide [1,2]. This disease remains a significant health challenge in developing countries, such as India, particularly because it increases the risk of human immunodeficiency virus (HIV) infection [3,4]. Accurate diagnostic tests are crucial for effective syphilis control.

Diagnosticians typically use serological tests for syphilis, which detect either non-treponemal antibodies, such as rapid plasma reagin (RPR) and venereal disease research laboratory (VDRL) tests, or *Treponema*-specific antibodies, such as the *T. pallidum* hemagglutination assay (TPHA), enzyme immunoassays (EIA), chemiluminescence immunoassays (CIA), *T. pallidum* passive particle agglutination test, and rapid treponemal assays. A single serological test, whether non-treponemal or treponemal, is insufficient for diagnosing syphilis. For example, non-treponemal tests can yield false negatives during the primary and late stages of infection or false positives due to HIV, autoimmune conditions, vaccinations, injecting drug use, pregnancy, or older age. The Centers for Disease Control and Prevention recommends confirming a reactive non-treponemal test with a treponemal test [5].

Non-treponemal tests are cost-effective, widely available, and simple to administer. They also allow for monitoring treatment response as titers decrease post-treatment. However, these tests may miss early primary and late syphilis due to their lower sensitivity and the possibility of a prozone reaction, which can also lead to biological false positives [5].

Some clinical laboratories and blood banks now use treponemal tests, such as EIA or CIA, for screening [6-8]. If these tests are reactive, a non-treponemal test like RPR is performed. If the non-treponemal test is non-reactive, a second treponemal test confirms the initial results and aids in patient management. This reverse screening algorithm detects previously treated, untreated, or incompletely treated syphilis cases and identifies individuals with false-positive results who are unlikely to have the disease. While the reverse algorithm is more sensitive for detecting early and latent syphilis and is objective, it is technically challenging, more costly, and unsuitable for monitoring treatment response. Treponemal reactivity often persists throughout a patient's life [5].

Some studies frequently report discordant results, where initial tests are negative, but subsequent tests show reactivity. Such discrepancies create diagnostic challenges for healthcare providers and cause undue patient stress. In cases of discordant results, further tests may be necessary to clarify diagnoses [8-10]. This study aims to evaluate the detectability of syphilis using commercially available non-treponemal and treponemal tests with serum samples from the Antenatal Clinic (ANC) and the Sexually Transmitted Infection (STI) Clinic at North Eastern Indira Gandhi Regional Institute of Health and Medical Sciences (NEIGRIHMS), a tertiary care hospital in Northeast India. This study was undertaken as part of a quality improvement effort to enhance syphilis serology accuracy and ensure the timely delivery of precise patient reports by evaluating commercially available test kits.

## Materials and methods

We conducted a prospective study from January 2019 to December 2021 after obtaining ethical clearance from the Institutional Ethical Committee (IEC) of North Eastern Indira Gandhi Regional Institute of Health and Medical Sciences (NEIGRIHMS) (approval number: P-301/15/082). The study involved 2312 samples, including 2124 from the ANC in the Department of Obstetrics and Gynaecology and 188 from the STI Clinic in the Department of Dermatology. We tested all samples using four serological tests: the non-treponemal RPR test (Carbogen, Tulip Diagnostics), the TPHA (Bio-Rad), TP-enzyme-linked immunosorbent assay (ELISA; Erba Lisa 96T, Transasia Bio-Medicals Ltd.), and Syphicheck WB (Tulip Diagnostics). The RPR test uses a carbon particulate suspension coated with lipid complexes to detect anti-lipoidal antibodies. The TPHA kits employ preserved avian erythrocytes coated with *T. pallidum* (Nichol's strain) antigens, which bind to specific antibodies in the patient's serum or plasma. The TP-ELISA is designed for the qualitative detection of total antibodies (immunoglobulin (Ig) M (IgM), IgA, and IgG) against *T. pallidum*. Syphicheck WB is a rapid immunochromatographic test that detects treponemal antibodies against recombinant *T. pallidum* antigens (47kDa, 17kDa). We performed and interpreted all tests following the manufacturer's instructions.

We analyzed the data using IBM SPSS Statistics for Windows, Version 21.0 (Released 2012; IBM Corp., Armonk, New York, United States). We assessed the agreement between the traditional and reverse algorithms for syphilis testing by calculating the percentage of agreement and Cohen's kappa (κ) value. Additionally, we determined the agreement between the TPHA and the modified TPHA test. We interpreted the strength of Cohen's κ agreement as follows: poor (<0.00), slight (0.00-0.20), fair (0.21-0.40), moderate (0.41-0.60), substantial (0.61-0.80), and almost perfect (0.81-1.00) [11]. We also calculated the sensitivity, specificity, positive predictive value, negative predictive value, and accuracy of the four tests in clinically diagnosed cases of primary or secondary syphilis.

Our laboratory participates in the syphilis serology External Quality Assurance Scheme conducted by the Regional STI Training Research and Reference Laboratory under the National AIDS Control Organization to ensure external quality assurance. Additionally, we performed internal quality checks using known positive (low positive) and negative control samples with each test batch.

## Results

**Figure 1:**
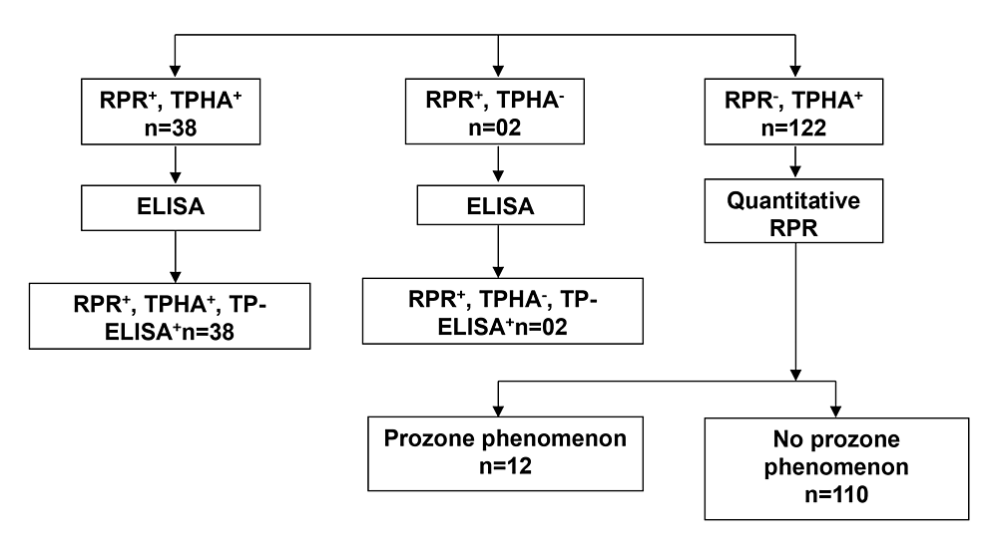
Testing using RPR, TPHA, and ELISA RPR: rapid plasma reagin; TPHA: *Treponema pallidum* hemagglutination assay; ELISA: enzyme-linked immunosorbent assay

We tested 2312 serum samples for syphilis, comprising 2124 from the ANC and 188 from the STI Clinic. Of these, 34 samples tested positive with all four tests: RPR (Carbogen), TPHA (Bio-Rad), TP-ELISA (Erba Lisa 96T), and Syphicheck WB (Tulip). We found 2098 samples negative by all tests. One hundred and eighty samples tested positive by one, two, or three tests. Concurrent RPR and TPHA positivity occurred in 38 samples (18 from the STI Clinic and 20 from the ANC), and all 38 were also positive by ELISA (Figure 1).

The seroprevalence of syphilis, based on RPR and TPHA positivity, was higher in the STI Clinic (9.6%, 18/188) compared to the ANC (0.9%, 20/2124). Two samples were RPR positive but TPHA negative, yet both were ELISA positive. Another 122 samples were negative for RPR but positive for TPHA; 58 were reactive by TP-ELISA, and 64 were non-reactive. We further tested the 122 RPR non-reactive samples by serum dilution to detect the prozone phenomenon; only 12 samples (0.5%) tested positive for RPR after dilution, which were also positive by TP-ELISA.

Among the 2312 patients, 102 were clinically diagnosed with syphilis (44 from the STI Clinic and 58 from the ANC; Table 1).

**Table 1:** Comparison of four serological tests against the clinical diagnosis PPV: positive predictive value; NPV: negative predictive value; RPR: rapid plasma reagin; TPHA: *Treponema pallidum* hemagglutination assay; ELISA: enzyme-linked immunosorbent assay; CI: confidence interval

Test	Clinical diagnosis (n=102)		Sensitivity (95%CI)	Specificity (95%CI)	PPV	NPV	Accuracy
	Positive	Negative					
RPR	Positive		39.22% (26.69-49.38%)	100% (99.83-100%)	100% (91.19-100%)	97.27% (96.83-97.66%)	97.32% (96.58-97.94%)
	40	0					
	Negative						
	62	2210					
TPHA	Positive		96.08% (90.26-98.92%)	97.19% (96.42-97.84%)	61.25% (55.21-66.96%)	99.81% (99.52-99.93%)	97.15% (96.38-97.79%)
	98	62					
	Negative						
	4	2148					
ELISA	Positive		96.08% (90.26-98.92%)	99.10% (98.61-99.45%)	83.05% (75.97-88.36%)	99.82% (99.52-99.93%)	98.96% (98.46-99.33%)
	98	20					
	Negative						
	4	2190					
Modified TPHA (Syphicheck)	Positive		68.63% (58.69-77.45%)	98.37% (97.75-98.86%)	66.04% (57.82-73.39%)	98.55% (98.08-98.91%)	97.06% (96.29-97.71%)
	70	36					
	Negative						
	32	2174					

The TPHA and TP-ELISA tests showed the highest sensitivity at 96.08% (95% confidence interval (CI), 90.26-98.92%), with a high negative predictive value of 99.81% (95%CI, 99.52-99.93%) for TPHA and 99.82% for TP-ELISA (95%CI, 99.69-49.38%). RPR demonstrated lower sensitivity (39.22%, 95%CI, 26.52-52.69%) but the highest specificity of 100% (95%CI, 99.83-100%). Both TPHA and modified TPHA showed lower positive predictive values of 61.25% (95%CI, 55.21-66.96%) and 66.04% (95%CI, 57.82-73.39%), respectively, compared to other tests. The percentage of agreement between TPHA and modified TPHA was 94.6%, with a κ value of 0.49 (95%CI, 0.42-0.57), interpreted as moderate agreement (Table 2). The strength of agreement between the traditional and reverse algorithms was also moderate, with a 97.3% agreement and a κ value of 0.53 (95%CI, 0.44-0.64; Table 3).

**Table 2:** Comparison of TPHA versus modified TPHA CI: confidence interval; TPHA: *Treponema pallidum* hemagglutination assay

Test	Modified TPHA (Syphicheck)		Total	Agreement (%)	κ value (95%CI)
TPHA (Bio-Rad)	Positive	Negative			
Positive	70	90	160	94.6%	0.49 (0.42330-0.57403)
Negative	36	2116	2152		
Total	106	2206	2312		

**Table 3:** Comparison of traditional versus reverse algorithm CI: confidence interval

Algorithm	Reverse algorithm		Total	Agreement (%)	κ value (95%CI)
Traditional algorithm	Positive	Negative			
Positive	38	0	38	97.3%	0.53 (0.43943-0.64010)
Negative	62	2212	2274		
Total	100	2212	2312		

## Discussion

This study evaluated the performance of treponemal and non-treponemal tests for syphilis and established the ideal testing algorithm for our laboratory based on the results. We found that 122 samples were non-reactive with the RPR test but tested positive for the TPHA (Bio-Rad). Upon retesting the RPR-negative samples using serum dilutions, we observed a prozone phenomenon in 12 (0.5%) cases (Figure 1). Of the remaining 110 samples, 76 also reacted to TP-ELISA. The prozone phenomenon, where high antibody titers in conditions such as secondary or early latent syphilis can lead to false-negative non-treponemal test results, highlights a limitation of these tests, especially their lack of sensitivity in early primary and late syphilis stages [12,13].

Our findings highlight the issues with the traditional syphilis serodiagnosis algorithm, which relies on non-treponemal tests like VDRL and RPR. Although these studies suggest a correlation with the disease, we found that using RPR alone to screen 102 clinically diagnosed patients with primary or secondary syphilis resulted in low sensitivity despite a 100% specificity. This highlights the risk of missed diagnoses, particularly in screening pregnant women.

However, treponemal antibody tests, such as TPHA (Bio-Rad) and TP-ELISA, demonstrated significantly higher sensitivity and specificity. TPHA and TP-ELISA showed the highest sensitivities and high negative predictive values [14-16]. TP-ELISA, which detects total antibodies (IgM, IgA, and IgG), has been shown in some studies to have higher sensitivity compared to RPR, although treponemal IgM results require careful interpretation due to potential lack of specificity [14,17,18].

The specificity of TP-ELISA was high in our study. Previous studies using EIA have demonstrated similar specificity levels [19]. However, syphilis diagnosis should not rely solely on EIA results but should be interpreted alongside other treponemal and non-treponemal test results and clinical correlations. We found that the positive predictive values were lowest in TPHA and modified TPHA. The agreement between TPHA and modified TPHA was moderate.

Our study confirmed the reverse algorithm's effectiveness, capturing more cases than the traditional algorithm due to the greater sensitivity of EIA tests. All RPR-positive cases were reactive to TPHA and TP-ELISA but not vice versa, demonstrating the superior sensitivity of treponemal tests. However, non-treponemal tests showed higher specificity. Despite a higher false-positive rate reported in some studies [20], the traditional algorithm is still suitable for low-volume laboratories.

No single test or definitive reference standard exists that can detect all stages of syphilis across different populations. Most tests are simple to perform, but without clinical signs and symptoms, interpreting serological test results becomes challenging. Thus, syphilis diagnosis should rely on both treponemal and non-treponemal methods to guide patient management decisions. Local disease prevalence, laboratory volume, cost, and staff should also be considered before implementing an algorithm to enhance syphilis prevention and control. Given our laboratory's low volume load and the sensitivity and specificity of the various tests, screening samples using RPR and TPHA is recommended.

This study has several limitations that warrant consideration. First, the sample size, though substantial, is drawn from a specific geographical region and healthcare setting, which may limit the generalizability of our findings to other populations and regions with different epidemiological profiles of syphilis. Additionally, the study relies on the performance of commercially available test kits, whose results might vary with manufacturer-specific reagents and methodologies, potentially affecting the reproducibility of our results in settings using different brands or types of tests. Another limitation is the study's observational design, which can introduce biases related to case selection and data collection. Moreover, we did not explore the potential impact of demographic variables such as age, gender, and comorbid conditions, which could influence the serological response and the performance of the tests. Lastly, interpreting some results, particularly those involving the prozone phenomenon and low predictive values, may require more sophisticated analytical techniques to understand the underlying dynamics and improve diagnostic algorithms fully.

## Conclusions

We conducted this study as part of a quality improvement initiative aimed at enhancing the quality of syphilis serology and ensuring accurate and timely patient reports through evaluating commercially available test kits. While the EIA can reduce the seronegative window following infection, we found that this method is time-consuming, requires specialized expertise, and is not feasible for daily syphilis testing. In populations with a low prevalence of syphilis, treponemal tests like EIA, which have lower specificity, could lead to false-positive results, causing unnecessary harm and emotional distress due to incorrect diagnoses. Therefore, our laboratory recommends screening samples using both the RPR and the TPHA to diagnose and confirm syphilis accurately. This combined testing approach is easier to perform, more familiar, faster, and less costly.
